# A Theoretical Study on the Spatiotemporal Variation in the Temperature Field in Linings of High-Water-Temperature Tunnels

**DOI:** 10.3390/ma16227139

**Published:** 2023-11-13

**Authors:** Mingli Huang, Meng Huang, Jiacheng Li, Yuan Qian

**Affiliations:** 1 School of Civil Engineering, Beijing Jiaotong University, Beijing 100044, China; mlhuang@bjtu.edu.cn (M.H.); 21115019@bjtu.edu.cn (J.L.); 2 College of Civil Engineering, Tongji University, Shanghai 200092, China; qianyuan678@tongji.edu.cn

**Keywords:** high-water-temperature tunnel, unsteady heat conduction, secondary lining, temperature field, concrete hydration heat, discrete equation

## Abstract

On the basis of the theory of unsteady heat conduction, discrete equations for the unsteady temperature field in the secondary linings of high-water-temperature tunnels when considering the hydration heat of lining concrete were derived and established. Spatiotemporal variation in the temperature field of tunnel linings was revealed through the analysis of numerical examples. Research demonstrates that the temperature of the secondary lining within a thickness range of approximately 15 cm near the tunnel clearance decreases sharply under the condition that the lining thickness is 35 cm. The higher the temperature on the lining’s outer surface, the more drastically the lining temperature decreases. When considering the hydration heat of lining concrete, the lining temperature increases to a certain extent after a sudden drop, reaching stability after approximately 20 h, and the lining temperature is approximately 1–2 °C higher than that without taking concrete hydration heat into account. The temperature difference between the tunnel lining’s core and its inner and outer surfaces is positively and negatively correlated with the temperature of the secondary lining’s outer surface, respectively. When the temperature of the secondary lining’s outer surface is not higher than 65 °C, the temperature difference between the tunnel lining’s core and its inner and outer surfaces is less than 20 °C. Conversely, it partially or completely exceeds 20 °C, in which case an insulation method is recommended to utilize to prevent thermal cracks in secondary linings triggered via a high temperature difference.

## 1. Introduction

In the context of the vigorous development of railways in China, tunnel projects have been increasingly and regularly constructed in high-temperature regions in recent years. Engineering diseases triggered via elevated temperatures have gradually become increasingly outstanding. As an example, six tunnels in the Lari railway are subjected to heat damage begotten via elevated ground temperatures, with the temperature of rock and water up to 80 °C. High ground temperatures can worsen the construction environment and affect the mechanical properties [1] and durability of concrete. Methods to solve this problem include developing and applying high-performance materials [2], setting up insulation layers [3], adopting air layer structures [4], and strengthening ventilation and cooling in tunnels [5]. Moreover, the thermal stress caused via high temperatures can also cause cracks in the tunnel lining and deformation of surrounding rock, etc. [6], posing a threat to the construction safety and performance of tunnel linings [7]. Currently, installing thermal insulation layers has been recognized as an efficacious measure to reduce the heat-exchanging efficiency between the tunnel lining and surrounding rock [8]. There are many studies on the tunnel problems in cold regions, but relatively limited research on high-temperature tunnels. Due to the delayed research on high-temperature tunnels in China, the relevant norms or standards are still incomplete. Therefore, it is urgent to accelerate the research on the issues related to the high-temperature tunnels.

Temperature significantly affects the hydration reaction of concrete, thereby affecting its early and long-term performance. At present, there are many related studies on the influence of high temperatures on the hydration reaction of concrete materials. However, there is limited research on the impact of high temperatures, especially high water temperatures, on the temperature field of tunnel linings. Additionally, the study of the temperature field of tunnel linings under high temperatures can lay the foundation for the study of the influence of high temperatures on the hydration of lining concrete. In terms of the impact of high temperatures on the hydration reaction of concrete, Sinno et al. [9] investigated the effect of temperature on alkali release from aggregates, alkali leaching from concrete samples, and the hydration and alkali-binding capacity of SCM. Research found that aggregates contribute more alkalis to alkaline solutions at higher temperatures. Kim et al. [10] analyzed the effect of temperature on concrete hydration using experimental and theoretical methods. Research indicates that high temperatures can increase the degree of early hydration reactions of materials such as cement in concrete, making its microstructure dense at an early age. Shirani et al. [11] found, through experimental research, that for ordinary Portland cement, its mechanical properties gradually worsen with an increase in temperature. Zajac et al. [12] found, through research, that high temperatures can result in densification of the matrix, which limits further reaction progress. Strangfeld et al. [13] studied the effect of high temperatures on the degree of concrete hydration and proposed a laboratory method for testing the degree of hydration. Farqad et al. [14] analyzed the effect of high temperatures on the hydration rate of cement pastes. Research indicates that cement pastes obtained at a high temperature had a faster densification than those of pastes obtained at a low temperature because of their higher rates of hydration. Tran et al. [15] conducted experiments on steam curing of cement paste and studied the effect of different high temperatures on the degree of the hydration of mixtures in cement paste. Leklou et al. [16] investigated the effect of temperature on a metakaolin–cement mortar blend from both macro- and microperspectives. Ghasabeh et al. [17] studied the influence of factors including temperature on thermal cracking during the hydration and hardening processes of concrete using a numerical calculation method.

In terms of the temperature field of tunnels, many studies have been systematically conducted. Lai et al. [18] adopted a dimensionless method to derive the temperature field solution during the freezing process of circular tunnels. Lu et al. [19,20] obtained the transient temperature field solution of circular tunnels through the analytical method of separating variables based on the superposition principle, which was further developed considering the temperature change with coordinates and convection at the boundary [21,22]. Toutain et al. [23] went deeply into the Laplace transform inversion of a heat transfer model. Li et al. [24] theoretically derived a formula for calculating and analyzing the characteristics of the temperature distribution in tunnel linings, while Liang et al. [25] also considered surrounding rock. Shao et al. [26] attained the temperature field solution in high-ground-temperature tunnels on the basis of a dimensionless method. Zhou et al. [27,28] derived the two-dimensional finite difference equation in tunnels on account of the theory of energy conservation and unsteady heat conduction, and provided the numerical solution for the temperature field in tunnels with elevated ground temperatures. Xu et al. [29] studied the spatial-temporal evolution principle of the temperature field in a high-temperature geothermal highway tunnel. Alhawat et al. [30] used the experimental method to study the thermal behavior of the tunnel lining under high temperatures.

In summary, the influence of concrete hydration heat on the lining temperature field is seldom paid attention to. However, concrete hydration heat, especially mass concrete, can have an undeniable impact on the lining temperature field. The impact of concrete hydration heat has been considered in individual studies, but not been directly taken into account in the process of theoretical derivation. Instead, the only proposed correction term has been supplied in the final calculation formula. Currently, the temperature field variation in the tunnel structure and surrounding rock are of great constant concern, while the spatiotemporal evolution of the lining temperature field of high-ground-temperature tunnels, especially high-water-temperature tunnels, has hardly received special attention. In view of this, a theoretical approach, directly considering the impact of concrete hydration heat on the basis of the theory of unsteady heat conduction, was utilized to construct discrete equations for the unsteady temperature field in the secondary linings of high-water-temperature tunnels. Through a comparative analysis of numerical examples, the spatiotemporal evolution of the lining temperature field was finally revealed. This study aims to lay a foundation for future research on the deterioration mechanism and characteristics of the tunnel lining under temperature stress and in situ stress. At the same time, it can also provide guidance for temperature control during the maintenance process of the secondary lining of high-water-temperature tunnels, thereby controlling the generation and propagation of lining cracks.

## 2. Analysis of the Lining Temperature Field of High-Water-Temperature Tunnels

Based on the theory of unsteady heat conduction, considering concrete hydration heat and high water temperatures, the mathematical expression for the spatiotemporal evolution of the lining temperature field in tunnels with a high water temperature is eventually established with time and space as control factors, providing a fundamental basis for subsequent research on maintenance technology of the secondary linings of high-water-temperature tunnels. It should be noted that the spatiotemporal variation in the temperature field refers to the temperature changes at different locations and times of tunnel linings under different high water temperatures when considering concrete hydration heat. Table 1 shows the symbols in the equations.

### 2.1. The Theory of Unsteady Heat Conduction

Periodic and non-periodic heat conduction constitute unsteady heat conduction. The former refers to periodic change in the object temperature over time, while the latter refers to the continuous increase or decrease in the object temperature over time. The object temperature gradually tends to medium temperature after some time, and ultimately reaches a thermal equilibrium. Obviously, heat conduction of tunnel linings is aperiodic and unsteady.

#### 2.1.1. The Conduction Differential Equation in the Unsteady State

The general form of the conduction differential equation in the unsteady state [18] in the Cartesian coordinate system can be expressed as Equation (1). From left to right in Equation (1), the first subitem is the increment of the thermodynamic energy of the microelement per unit time (the unsteady term). The subitem with parentheses in the middle refers to the energy that the microelement increases per unit time through heat conduction at the interface (diffusion term). Further, the remaining subitem represents the internal heat source (source term).

(1) $$\frac{\partial T}{\partial \tau }=\alpha \left(\frac{\partial^{2}T}{\partial x^{2}}+\frac{\partial^{2}T}{\partial y^{2}}+\frac{\partial^{2}T}{\partial z^{2}}\right)+\frac{\overset{•}{\Phi }}{\rho c}$$

where

*T*—The object temperature (°C), which is a function with variables *x*, *y*, *z* and *τ*.

*τ*—Time of heat conduction (s).

*α*—Thermal diffusivity (m^2^/s). $\alpha =\frac{\lambda }{\rho c}$, where *λ* is thermal conductivity (W/m·°C), *ρ* is medium density (kg/m^3^), and *c* is the specific heat of the medium (J/kg·°C).

$\overset{•}{\Phi }$—Internal heat source (source term), which refers to the energy released in unit time of the microelement.

Equation (1) can be expressed as polar coordinates, as shown in Equation (2).

(2) $$\frac{\partial T}{\partial \tau }=\alpha \left(\frac{\partial^{2}T}{\partial r^{2}}+\frac{1}{r}\frac{\partial T}{\partial r}+\frac{1}{r^{2}}\frac{\partial^{2}T}{\partial \theta^{2}}\right)+\frac{\overset{•}{\Phi }}{\rho c}$$

**Figure 1:**
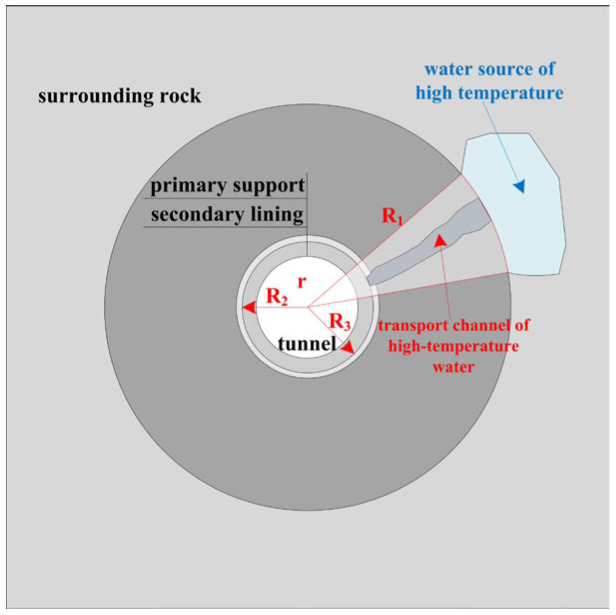
Schematic of the tunnel structure with elevated-temperature water.

Assuming the tunnel is circular and only conducts heat radially along the tunnel, simplify Equation (2) to obtain the one-dimensional heat conduction equation in the unsteady state as polar coordinates, as shown in Equation (3), where *r* is the radius and the other parameters are the same as above. Figure 1 shows a schematic of the tunnel structure with elevated-temperature water.

(3) $$\frac{\partial T}{\partial \tau }=\alpha \left(\frac{\partial^{2}T}{\partial r^{2}}+\frac{1}{r}\frac{\partial T}{\partial r}\right)+\frac{\overset{•}{\Phi }}{\rho c}$$

In previous studies, when analyzing the lining temperature field, the internal heat source of the differential equation for heat conduction in the unsteady state is not considered, or the obtained analytical solution is modified to take into account the internal heat source. Thus, the results obtained usually have certain limitations. As we all know, the secondary lining will release a large amount of heat in the process of pouring and curing due to concrete hydration, which significantly affects the lining temperature field, thus influencing the lining’s structural performance. Therefore, it is indispensable to take into account the internal heat source when studying the spatiotemporal evolution of the lining temperature field, and we utilize the finite difference method to obtain a solution that considers the internal heat source.

Assume that the secondary lining of the tunnel is divided into *k* layers along its thickness, and each layer has its own thermal parameters. The differential equation for the thermal conduction of the lining temperature field in the unsteady state considering the internal heat source is expressed as Equation (4).

(4) $$\frac{\partial T_{k}}{\partial \tau }=\alpha_{k}\left(\frac{\partial^{2}T_{k}}{\partial r^{2}}+\frac{1}{r}\frac{\partial T_{k}}{\partial r}\right)+\frac{{\overset{•}{\Phi }}_{k}}{\rho_{k}c_{k}}$$

where

*T_k_*—Temperature of the *k*-th layer of the lining (°C).

*α_k_*—Thermal diffusivity of the *k*-th layer of the lining (m^2^/s). $\alpha_{k}=\frac{\lambda_{k}}{\rho_{k}c_{k}}$. *λ_k_* is the thermal conductivity of the *k*-th layer of the lining (W/m·°C), *ρ_k_* is the density of the *k*-th layer of the lining (kg/m^3^), and *c_k_* is the specific heat of the *k*-th layer of the lining (J/kg·°C).

${\overset{•}{\Phi }}_{k}$—Internal heat source of the *k*-th layer of the lining.

*r*—Radius of the *k*-th layer of the medium (m).

#### 2.1.2. Boundary Conditions for Theoretical Derivation

To handle specific issues, corresponding boundary and initial conditions are essential to solve the differential equation for heat conduction. Boundary conditions adopted in this study are as follows.

(1) The first boundary condition: the temperature distribution on the boundary is known. For heat conduction in the unsteady state, it is expressed as Equation (5).

(5) T=f(τ)

(2) The second boundary condition: heat flux at the boundary is known. For heat conduction in the unsteady state, it is expressed as Equation (6), where ***n*** is the vector on the outer surface (the same below).

(6) $$-\lambda \frac{\partial T}{\partial n}=f\left(\tau \right)$$

(3) The third boundary condition: the coefficient of convectional heat transfer *h* and fluid temperature *T_f_* are known. For heat conduction in the unsteady state, it is expressed as Equation (7).

(7) $$-\lambda \frac{\partial T}{\partial n}=h\left(T-T_{f}\right)$$

### 2.2. Calculation of the Lining Temperature Field of High-Water-Temperature Tunnels

#### 2.2.1. Basic Assumptions

To establish the calculation equation for the spatiotemporal evolution of the lining temperature field in high-water-temperature tunnels considering concrete hydration heat, are made as follows.

(1) The tunnel lining is simplified to circular lining, and heat only radially transfers along the tunnel.
(2) The medium in each layer of tunnel linings meets the conditions of homogeneity and isotropy.
(3) The thermal parameters of tunnel linings (density, specific heat and heat conductivity, etc.) and air temperature inside the tunnel remain constant.
(4) Adjacent layers of tunnel linings are in close contact, and their heat flux and temperature have consecutive characteristics.

#### 2.2.2. Discrete Equation for the Unsteady Temperature Field in the Tunnel Lining

**Figure 2:**
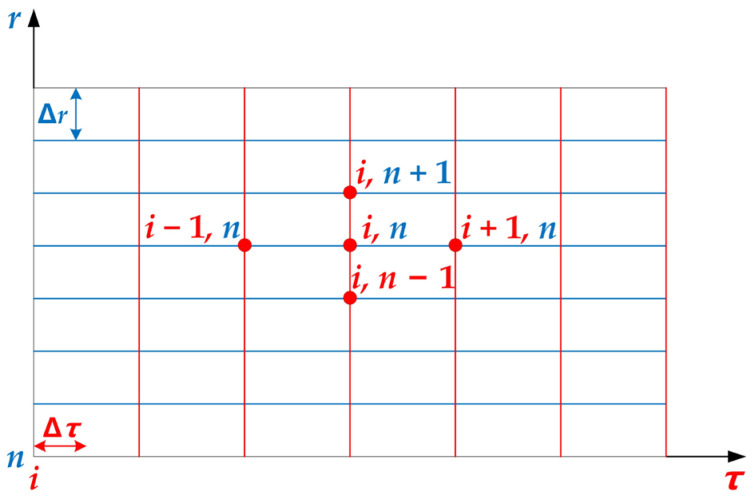
Schematic of the time–space discretization of the unsteady temperature field.

A finite number of sets of values at the discrete points are used to replace the continuous temperature fields in space and time. Then, through solving the algebraic equations established via a certain method regarding the above values, the values of the temperature to be calculated at the discrete points are finally obtained. The spatiotemporal zone of the unsteady temperature field can be discretized according to Figure 2. In Figure 2, *r* and *τ* represent spatial and temporal coordinates, respectively. Further, Δ*r* and Δ*τ* refer to spatial and temporal steps, respectively. Moreover, (*n*, *i*) represents the node position in the spatiotemporal zone, the temperature of which can be denoted as $T_{n}^{i}$.

There are two types of nodes in the calculation model. Internal nodes refer to nodes that are involved in heat conduction of secondary lining in the calculation region, while boundary nodes represent nodes that are involved in convective heat exchange on the lining’s internal boundary.

1. Establishment of internal node equations

The control equation shown in Equation (4) includes the diffusion terms for temperature in space $\frac{\partial^{2}T_{k}}{\partial r^{2}}$, $\frac{\partial T_{k}}{\partial r}$, the unsteady term for temperature in time $\frac{\partial T_{k}}{\partial \tau }$, and the internal heat source term $\frac{\overset{•}{\Phi_{k}}}{\rho_{k}c_{k}}$.

Discretization of the diffusion terms

(1) Write Taylor expansions (8) and (9) of the function t for the point (*n*, *i*), respectively.

(8) $$T_{n+1}^{i}=T_{n}^{i}+\Delta r\frac{\partial T}{\partial r}|{}_{n,i}+\frac{\Delta r^{2}}{2}\frac{\partial^{2}T}{\partial r^{2}}|{}_{n,i}+\frac{\Delta r^{3}}{6}\frac{\partial^{3}T}{\partial r^{3}}|{}_{n,i}+\ldots$$

(9) $$T_{n-1}^{i}=T_{n}^{i}-\Delta r\frac{\partial T}{\partial r}|{}_{n,i}+\frac{\Delta r^{2}}{2}\frac{\partial^{2}T}{\partial r^{2}}|{}_{n,i}-\frac{\Delta r^{3}}{6}\frac{\partial^{3}T}{\partial r^{3}}|{}_{n,i}+\ldots$$

Equation (10) can be calculated by adding Equations (8) and (9) and ignoring the Peano remainder in the Taylor expansion.

(10) $${\frac{\partial T^{2}}{\partial r^{2}}|}_{n,i}=\frac{T_{n+1}^{i}-2T_{n}^{i}+T_{n-1}^{i}}{\Delta r^{2}}$$

Equation (11) can be calculated by subtracting Equations (8) and (9) and ignoring the Peano remainder.

(11) $${\frac{\partial T}{\partial r}|}_{n,i}=\frac{T_{n+1}^{i}-T_{n-1}^{i}}{2\Delta r}$$

(2) Discretization of the unsteady state term

Equation (12) can be obtained via the Taylor expanding function *T*, as follows:

(12) $$T_{n}^{i+1}=T_{n}^{i}+\Delta \tau \frac{\partial T}{\partial \tau }|{}_{n,i}+\frac{\Delta \tau^{2}}{2}\frac{\partial^{2}T}{\partial \tau^{2}}|{}_{n,i}+\frac{\Delta \tau^{3}}{6}\frac{\partial^{3}T}{\partial \tau^{3}}|{}_{n,i}+\ldots$$

So, the unsteady term $\frac{\partial T_{k}}{\partial \tau }$ can be discretized into Equation (13) via omitting the Peano remainder.

(13) $${\frac{\partial T}{\partial \tau }|}_{n,i}=\frac{T_{n}^{i+1}-T_{n}^{i}}{\Delta \tau }$$

(3) Discretization of the internal heat source term

The concrete of tunnel linings emits heat during the hydration process after pouring, and hydration heat is regarded as the term for the internal heat source. In existing studies, empirical formulas for concrete hydration heat include the exponential, hyperbolic and composite exponential types. Currently, the composite exponential formula proposed by Zhu [31] is widely used, as shown in Equation (14).

(14) $$Q\left(\tau \right)=Q_{0}\left(1-e^{-a\tau^{b}}\right)$$

where *Q*(*τ*) is hydration heat when concrete age is *τ*, and its unit is kJ/kg. *Q*_0_ is the total heat released by cement hydration, and its unit is kJ/kg. *τ* refers to concrete age, and its unit is day; a and b are constant parameters, which can be set based on the cement grades and empirical coefficients. The concrete used in this study is C30 ordinary Portland cement, and the parameter values are based on the empirical formula for the heat release of concrete hydration summarized by Zhu [31], that is *Q*_0_ = 330 kJ/kg, *a* = 0.69, and *b* = 0.56.

**Figure 3:**
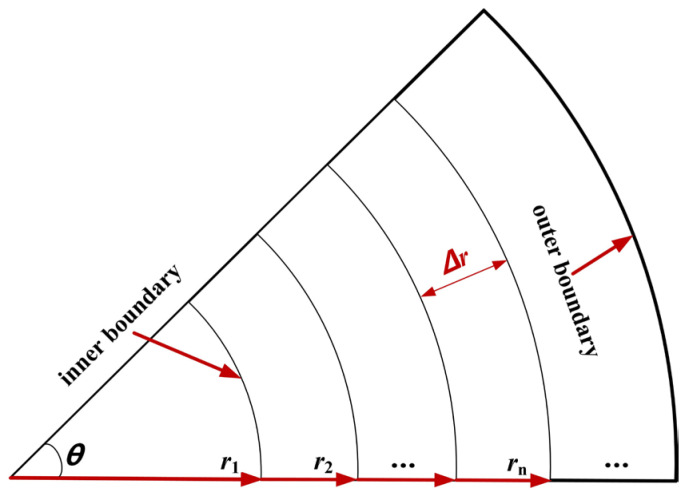
Calculation diagram of the internal heat source.

$\overset{•}{\Phi }$ refers to the increased energy of the microelement in unit time. Hydration heat is characterized as heat released by the hydration of the concrete microelement per unit time. Figure 3 shows the calculation diagram of the internal heat source. According to *Q*(*τ*), the heat released per unit time by the microelement at the node (*n*, *i*) can be expressed as Equation (15). Equation (16) can be obtained by determining Equation (15).

(15) $${\overset{•}{\Phi }|}_{n,i}=Q^{\prime }\left(\tau_{i}\right)\Delta \tau \rho r_{n}\theta \Delta r=abQ_{0}e^{-a{\left[\left(i-1\right)\Delta \tau \right]}^{b}}{\left[\left(i-1\right)\Delta \tau \right]}^{b-1}\Delta \tau \rho r_{n}\theta \Delta r$$

(16) $${\frac{\overset{•}{\Phi }}{\rho c}|}_{n,i}=\frac{abQ_{0}e^{-a{\left[\left(i-1\right)\Delta \tau \right]}^{b}}{\left[\left(i-1\right)\Delta \tau \right]}^{b-1}r_{n}\theta \Delta r\Delta \tau }{c}$$

(4) Discrete equation for internal nodes

Equations (10), (11), (13) and (16) are substituted into Equation (4), and the discrete equation for internal nodes can be obtained after determining Equation (17).

(17) $$T_{n}^{i+1}=F_{O}\left[\left(1+\frac{\Delta r}{2r_{n}}\right)T_{n+1}^{i}+\left(1-\frac{\Delta r}{2r_{n}}\right)T_{n-1}^{i}+\left(\frac{1}{F_{O}}-2\right)T_{n}^{i}\right]+\frac{abQ_{0}e^{-a{\left[\left(i-1\right)\Delta \tau \right]}^{b}}{\left[\left(i-1\right)\Delta \tau \right]}^{b-1}r_{n}\theta \Delta r\Delta \tau^{2}}{c}$$

where *i* = 1, 2, 3, …; *n* = 1, 2, 3, …

*F_O_*—Fourier number of grid. It is a dimensionless quantity used to describe the unsteady heat conduction and molecular diffusion, $F_{O}=\frac{\alpha \Delta \tau }{\Delta r^{2}}$.

*r_n_*—Vertical length from node (*n*, *i*) to the longitudinal axis of the tunnel (unit: m), *r_n_* = (*n* − 1)Δ*r*.

$T_{n}^{i}$—Temperature at node (*n*, *i*), K.

2. Establishment of boundary node equations
(1) Discrete equation for internal boundary nodes

**Figure 4:**
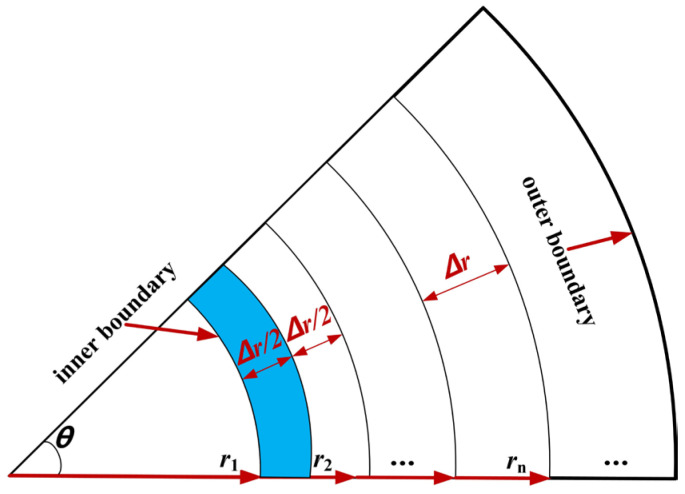
Diagram of convection heat exchange of boundary nodes.

On the basis of energy conservation, the discrete equation for internal boundary nodes is solved. The microelement ① with a width of $\frac{\Delta r}{2}$ is shown in the blue area in Figure 4. The left part adjacent to the microelement ① is the tunnel clearance, and the microelement ① conducts convection heat exchange with air in the tunnel. Microelement ① receives heat transmitted from microelement ② with a width of $\frac{\Delta r}{2}$ that is at the right side of microelement ①. In addition, microelement ① itself has the process of hydration heat that needs to be considered. Then, Equation (18) can be obtained by using the law of energy conservation for microelement ①.

(18) $$\lambda \frac{T_{2}^{i}-T_{1}^{i}}{\Delta r}\left(r_{1}+\frac{\Delta r}{2}\right)\theta +h\left(T_{1}^{i}-T_{f}\right)r_{1}\theta +Q_{0}\left\{1-e^{-a{\left[\left(i-1\right)\Delta \tau \right]}^{b}}\right\}\rho r_{1}\theta \frac{\Delta r}{2}=\rho cr_{1}\theta \frac{\Delta r}{2}\frac{T_{1}^{i+1}-T_{1}^{i}}{\Delta \tau }$$

By determining Equation (18), the discrete equation for the internal boundary nodes can be obtained, as shown in Equation (19).

(19) $$T_{1}^{i+1}=F_{O}\left[\left(2+\frac{\Delta r}{r_{1}}\right)T_{2}^{i}+2B_{i}T_{f}+\left(\frac{1}{F_{O}}-2B_{i}-\frac{\Delta r}{r_{1}}-2\right)T_{1}^{i}\right]+\frac{Q_{0}\left\{1-e^{-a{\left[\left(i-1\right)\Delta \tau \right]}^{b}}\right\}\Delta \tau }{c}$$

where *i* represents all integers greater than 0; *n* = 1.

*B_i_*—Resistance to the heat conduction ratio (Biot number). $B_{i}=\frac{2h\Delta \tau }{\rho c\Delta r}$ and *h* refer to the coefficient of convection conversion (unit: W/(m^2^·K)); *ρ* is the lining density (unit: kg/m^3^); *c* represents specific heat (unit: J/(kg·K)).

*T_f_*—Air temperature in the tunnel clearance (unit: K).

*r*_1_—Vertical length from the inner boundary to the tunnel’s longitudinal axis (unit: m).

(2) Discrete equation for the outer boundary nodes

In space, the outer boundary can be divided into nodes with a number of (*n* + 1). Since the outer boundary directly contacts the initial support boundary of the tunnel, when hot water with a high temperature reaches the lining’s outer surface through the migration channel, the temperature field of the lining’s outer boundary can be regarded as a constant temperature field, which is recorded as *T*_outer_, and the discrete equation for the outer boundary nodes can be expressed as Equation (20).

(20) $$T_{n+1}^{i}=T_{\mathrm{outer}}$$

where *i* = 1, 2, 3, …; *n* = 1, 2, 3, …

For Equations (17), (19) and (20), the time unit for the part that considers hydration heat is day. Further, the time unit for the other parts is second. Therefore, it is necessary to unify the units of time in the equations during the calculation process. In the example section of this study, the time units of the calculation equations are unified as hours.

#### 2.2.3. Solution Method for the Temperature Field in the Unsteady State

The following shows the calculation process for solving the temperature field in the unsteady state of the secondary linings of high-water-temperature tunnels.

(1) Set the basic assumptions, equate the tunnel to the circular tunnel, and establish heat conduction equations in the unsteady state as polar coordinates.
(2) Establish discrete equations for internal nodes, internal and external boundary nodes, and the internal heat source term.
(3) Select calculation parameters for secondary lining, including its dimensions, physical parameters, thermal parameters, boundary conditions for solving discrete equations for internal nodes, internal and external boundary nodes, and the internal heat source term, the spatial and temporal steps of the calculation and the calculation period.
(4) Use mathematical calculation software and language programming to iteratively solve the discrete equations for the internal nodes, internal and external boundary nodes. The new value at any time is solved based on the initial value of node temperature, which can be obtained through iterative calculation until the result converges. To ensure the stability and convergence of iterative computation, both the temporal and spatial steps need to satisfy Equations (21) and (22).

(21) $$F_{O}=\frac{\alpha \Delta \tau }{\Delta r^{2}}\leq \frac{1}{2}$$

(22) $$F_{O}\leq \frac{1}{2\left(1+B_{i}+\frac{\Delta r}{2r_{1}}\right)}$$

## 3. Example Analysis

Taking a circular cross-sectional tunnel as an example, the numerical analysis was carried out. A distinctive feature of high-water-temperature tunnels is that the source of heat refers to the elevated-temperature water, the temperature of which is considered constant. Therefore, when the lining’s outer surface encounters hot water flowing through the seepage path, its temperature is considered constant as well, which is different from high-rock-temperature tunnels.

### 3.1. Calculation Conditions and Parameters

#### 3.1.1. Setting of Working Conditions

The excavation radius, the size of the secondary lining and the material properties of the tunnel are as follows:

(1) Size of the calculation model: the excavation radius and secondary lining thickness of the circular tunnel are taken as 3.3 m and 0.35 m, respectively; the material of the lining concrete: C30 concrete; temperature on the lining’s inner boundary: 28 °C.
(2) Temperature on the lining’s outer boundary: assuming that the hot water with a high temperature from a certain heat source around the tunnel reaches the lining’s outer surface (near surrounding rock side). The high temperature directly acts on the secondary lining and has a direct impact on its temperature field. This is an extreme condition, and calculating the spatiotemporal evolution of the lining’s temperature field under this extreme condition is of great significance for the exploration and development of high-temperature curing techniques for tunnel linings. The working conditions are shown in Table 2.

#### 3.1.2. Calculation Parameters

In the example analysis, the secondary lining concrete of the tunnel is C30 concrete, and the calculation parameters of concrete and air (Table 3) are selected in line with the Code for Design of Railway Tunnel (TB 10003-2016) [32], together with the Code for Design of Concrete Structures (GB 50010-2010) [33].

### 3.2. Calculation Results

Substitute the calculation parameters in Table 3 with the derived discrete equations for the lining’s temperature field in the unsteady state in high-water-temperature tunnels, and use mathematical calculation software to program to calculate the spatiotemporal changes in the lining’s temperature field under each calculation condition.

#### 3.2.1. Calculation Results Considering Concrete Hydration Heat

**Figure 5:**
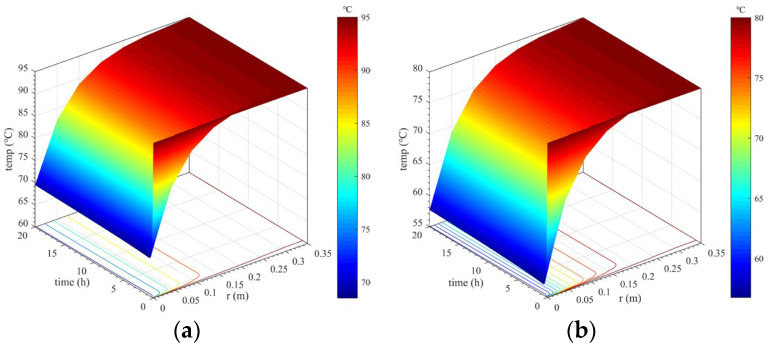

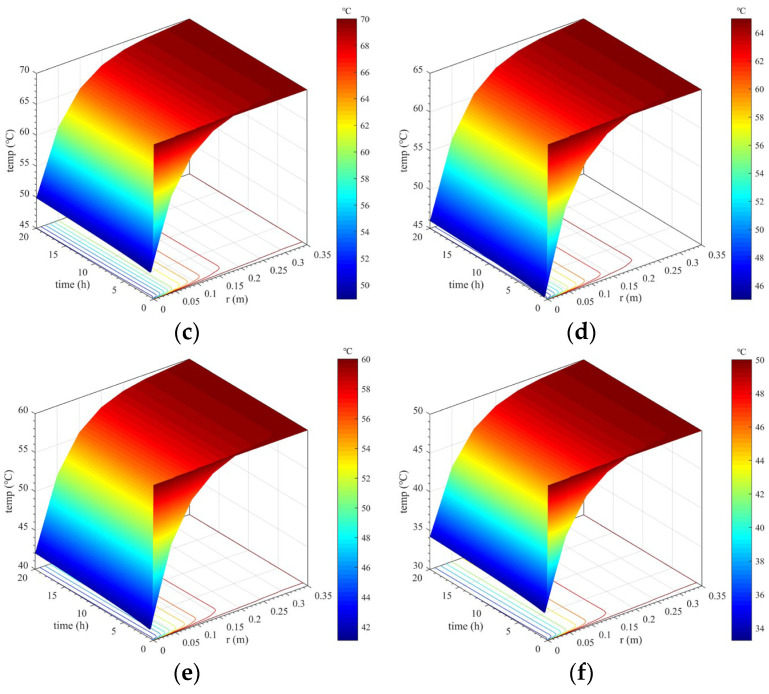
Spatiotemporal changes in the lining’s temperature field considering hydration heat. (**a**) *T*_outer_ = 95 °C; (**b**) *T*_outer_ = 80 °C; (**c**) *T*_outer_ = 70 °C; (**d**) *T*_outer_ = 65 °C; (**e**) *T*_outer_ = 60 °C; (**f**) *T*_outer_ = 50 °C.

**Figure 6:**
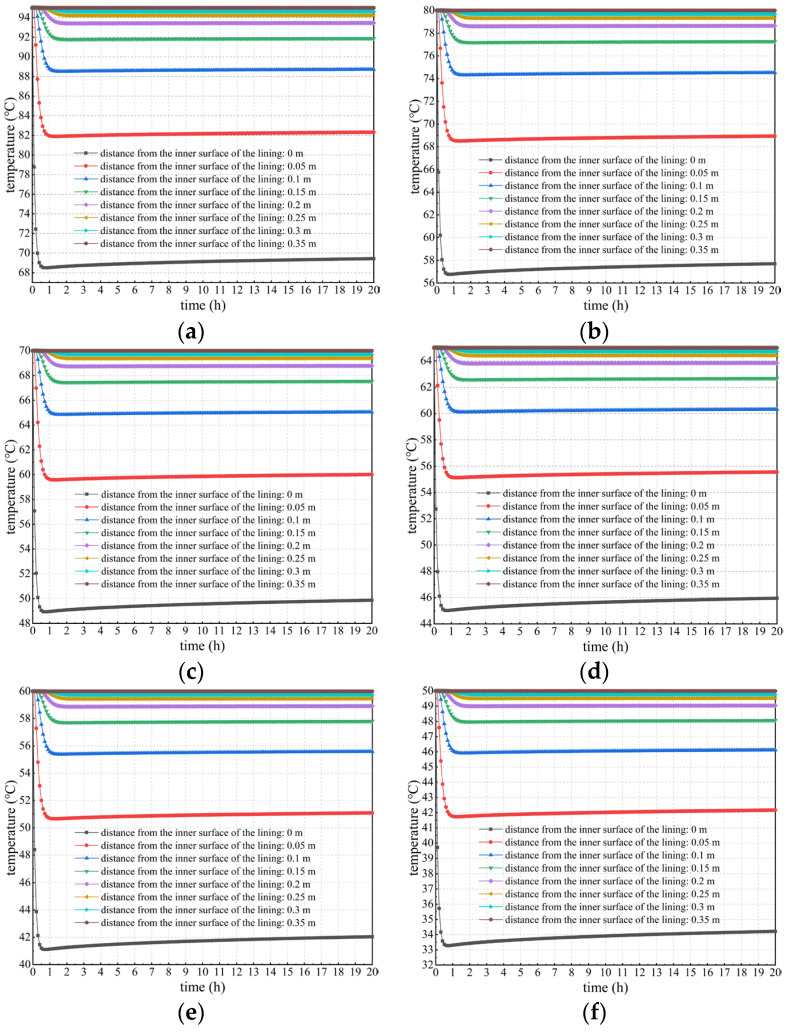
Temperature change curves of secondary lining at different temperatures and positions considering concrete hydration heat. (**a**) *T*_outer_ = 95 °C; (**b**) *T*_outer_ = 80 °C; (**c**) *T*_outer_ = 70 °C; (**d**) *T*_outer_ = 65 °C; (**e**) *T*_outer_ = 60 °C; (**f**) *T*_outer_ = 50 °C.

**Figure 7:**
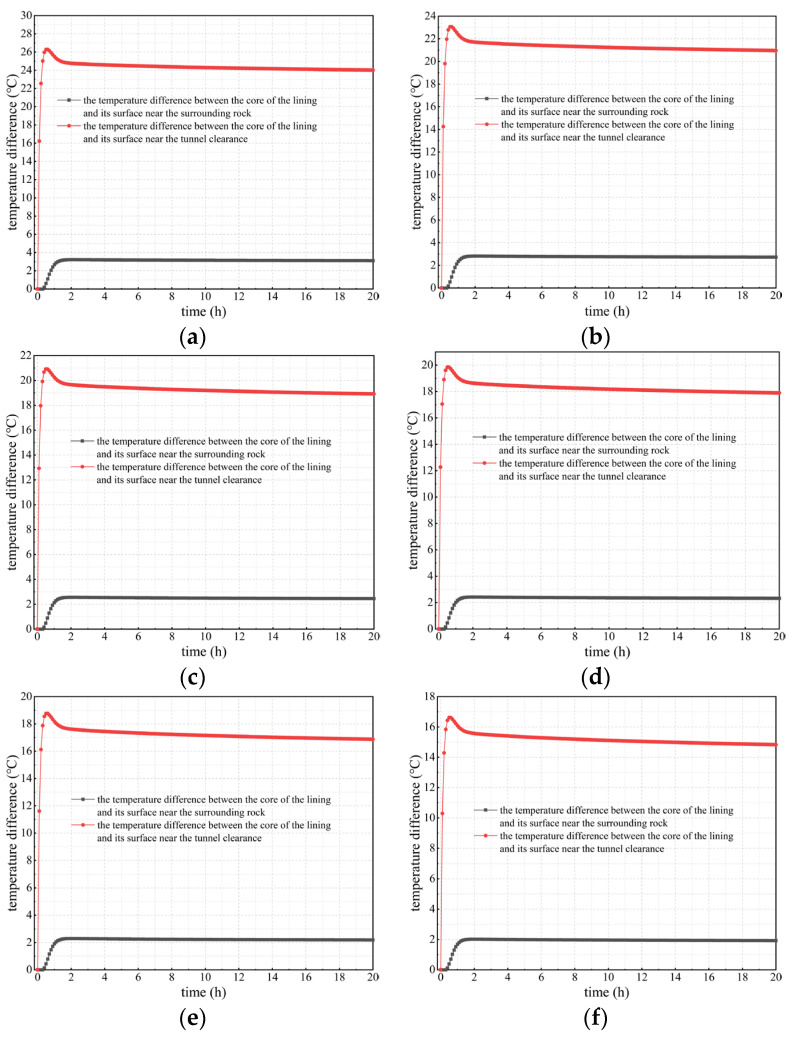
Curves of the temperature difference between the lining’s core and surfaces considering hydration heat. (**a**) *T*_outer_ = 95 °C; (**b**) *T*_outer_ = 80 °C; (**c**) *T*_outer_ = 70 °C; (**d**) *T*_outer_ = 65 °C; (**e**) *T*_outer_ = 60 °C; (**f**) *T*_outer_ = 50 °C.

Considering the heat release of concrete, the temperature changes in lining concrete at different positions and times were studied and analyzed, as well as the temperature difference between the lining core and its surfaces. Calculation results under different calculation conditions considering concrete hydration heat are shown in Figure 5, Figure 6 and Figure 7.

1. Spatiotemporal variation in the lining’s temperature field

Spatiotemporal variation in the lining’s temperature field under each calculation condition considering concrete hydration heat is shown in Figure 5. From an overall perspective, when considering concrete hydration heat, spatiotemporal changes in the lining temperature under various calculation conditions present similar laws. The closer the secondary lining is to the tunnel clearance side, the more rapidly the lining temperature decreases. Further, the higher the temperature of the lining’s outer surface, the more sharply the decrease rate increases. However, variation in the lining temperature closer to surrounding rock is smaller because the temperature of the lining’s outer surface remains constant, which continuously provides heat to the secondary lining. In addition, the lining temperature within a thickness range of approximately 15 cm near the tunnel clearance side decreases most sharply, and thus can be considered as a strong temperature change zone. Such a large temperature change can cause drastic changes in the temperature difference between the lining’s core and surfaces. This is extremely unfavorable to the lining concrete’s growth.

2. Variation in the lining temperature under different temperatures and locations

Figure 6 shows the curves of temperature versus time considering concrete hydration heat is plotted to specifically explore the spatiotemporal changes in the lining’s temperature field. Seen more intuitively and clearly from Figure 6, the temperature of secondary lining within a thickness of approximately 15 cm near the tunnel clearance decreases most sharply. The higher the temperature of the lining’s outer surface, the more drastically the temperature decreases. For example, when the temperature of the lining’s outer surface is 50 °C, the temperature at the lining’s inner surface decreases by approximately 15.8 °C after the temperature field reaches a stable level. However, when the outer surface temperature of secondary lining is 95 °C, its inner surface temperature decreases by approximately 25.6 °C after the temperature field reaches stability, which is approximately 62.0% more than that of 15.8 °C.

The depth of approximately 15 cm near the tunnel clearance is regarded as the boundary line. When the depth is greater than 15 cm, the lining temperature decreases and reaches equilibrium within approximately 2 h. After that, it remains basically stable and is not influenced by concrete hydration heat. In addition, the temperature after stabilization does not significantly differ from the temperature of the lining’s outer surface. This is because lining concrete near surrounding rock is significantly affected by the outer surface temperature of secondary lining. Concrete hydration heat within this range is relatively lower than heat transmitted from the lining’s outer surface, the contribution of which to temperature changes in secondary lining can be ignored. When the depth is less than 15 cm, the lining temperature rapidly decreases within 1.5 h due to the impact of ventilation and cooling measures. However, the lining temperature rises for some time after a sudden drop, reaching stability after approximately 20 h; and the closer it is to the inner surface of the lining, the more significant this phenomenon becomes. The reason for this is that the heat loss of the lining within this range is fast under the action of ventilation measures, while the contribution of hydration heat to changes in the lining temperature within this range gradually becomes prominent, and the lining temperature rises to a certain extent.

3. The temperature difference variation between the lining’s core and surfaces

The temperature difference between the lining’s core and inner and outer surfaces is an important indicator to determine whether microdamage such as cracks will occur in the curing process of the secondary lining concrete. The Code for Durability Design on Concrete Structure of Railway (TB 10005-2010) [34] states that, during the curing period of lining concrete, if the temperature difference mentioned above is greater than 20 °C, damage to the lining structure, such as thermal cracks, can be easily caused and this has an adverse effect on its performance and durability.

Figure 7 indicates that when considering concrete hydration heat, as the outer surface temperature of secondary lining increases, the absolute value of the temperature difference between the lining’s core and its outer surface shows a downward trend, but its decline is not significant. Under various calculation conditions, the temperature difference remains at 2–3 °C, which reflects the conclusion obtained earlier in this study that the temperature difference in secondary lining adjacent to surrounding rock is not significant. However, the absolute value of the temperature difference between the lining’s core and its inner surface (surface adjacent to the tunnel clearance) is positively correlated with the outer surface temperature of secondary linings. Under any calculation conditions, the temperature difference gradually decreases after a sharp increase of 2 h and reaches stability after approximately 20 h. This is due to the prominent impact of concrete hydration heat on changes in the lining temperature. When the outer surface temperature of secondary lining is 95 and 80 °C, the temperature difference between the lining’s core and its inner surface exceeds 20 °C. When the outer surface temperature is 70 °C, a temperature difference greater than 20 °C occurs within 2 h. When the outer surface temperature is 65, 60, and 50 °C, the temperature difference is less than 20 °C. Thus, the outer surface temperature of tunnel linings should not be higher than 65 °C; and at this time, the temperature of the core of the lining should not exceed 63 °C, consistent with the Code for Durability Design on Concrete Structure of Railway (TB 10005-2010) [34], which states that the core temperature of the secondary lining should not exceed 65 °C. Therefore, in the curing process of lining concrete, it is essential to control the outer surface temperature of secondary lining so as not to exceed 65 °C to ensure that the temperature difference between the lining’s core and surfaces is lower than the required 20 °C in the national standard mentioned above (TB 10005-2010) [34], thus preventing engineering problems such as lining cracking caused by a high temperature difference. To conclude, when the outer surface temperature of secondary lining exceeds 65 °C, alternative insulation measures need to be taken to reduce the adverse impact of the high temperatures of the lining’s outer surface during the curing period of lining concrete.

#### 3.2.2. Calculation Results When Not Considering Concrete Hydration Heat

**Figure 8:**
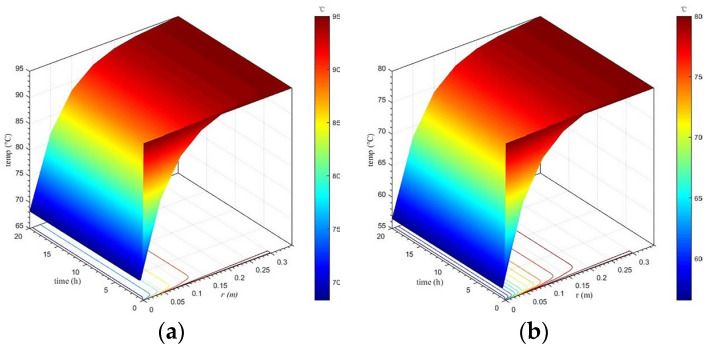

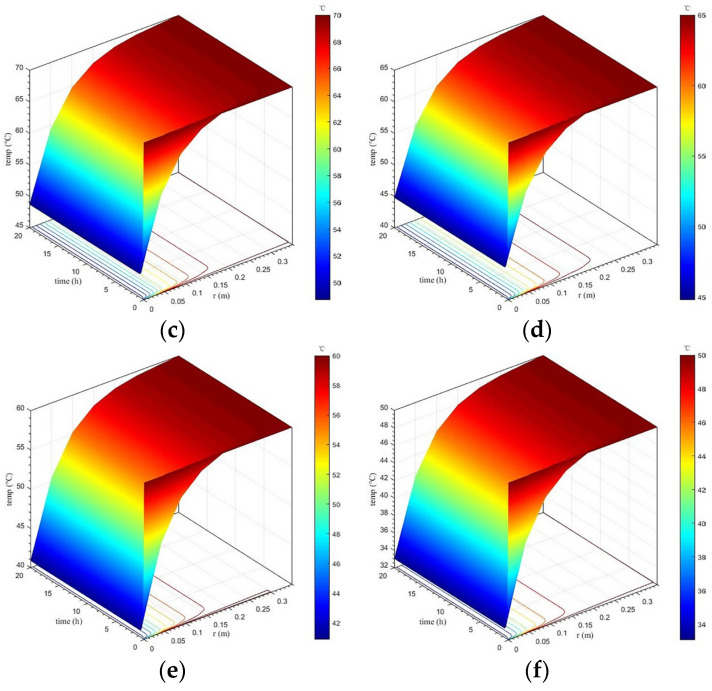
Spatiotemporal variation in the lining’s temperature field when not considering concrete hydration heat. (**a**) *T*_outer_ = 95 °C; (**b**) *T*_outer_ = 80 °C; (**c**) *T*_outer_ = 70 °C; (**d**) *T*_outer_ = 65 °C; (**e**) *T*_outer_ = 60 °C; (**f**) *T*_outer_ = 50 °C.

**Figure 9:**
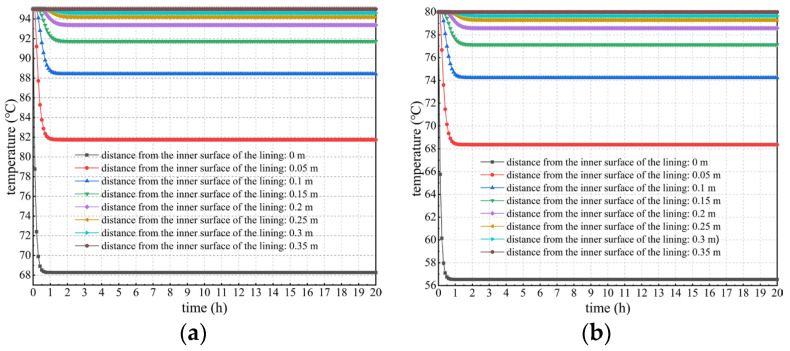

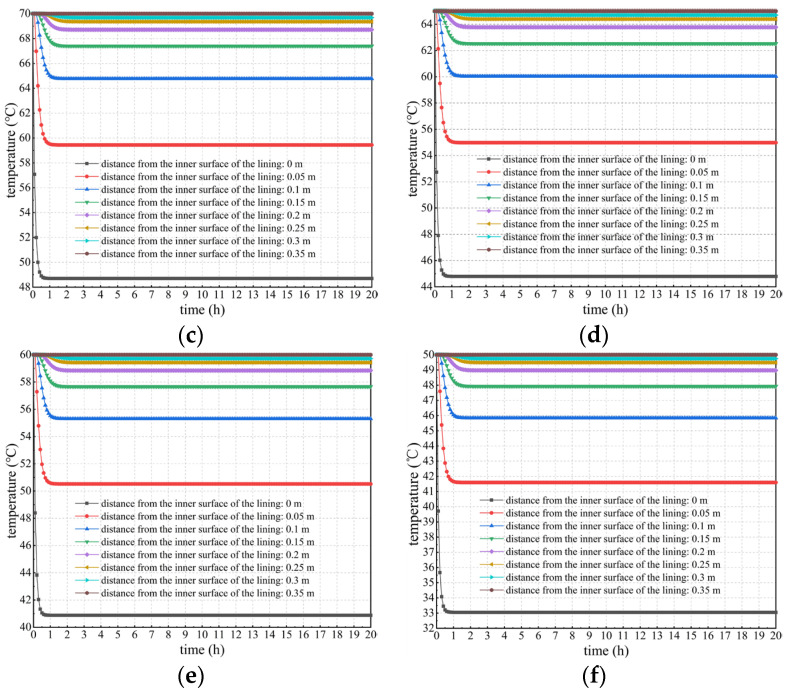
Changing curves of the lining temperature at different temperatures and positions when not considering concrete hydration heat. (**a**) *T*_outer_ = 95 °C; (**b**) *T*_outer_ = 80 °C; (**c**) *T*_outer_ = 70 °C; (**d**) *T*_outer_ = 65 °C; (**e**) *T*_outer_ = 60 °C; (**f**) *T*_outer_ = 50 °C.

**Figure 10:**
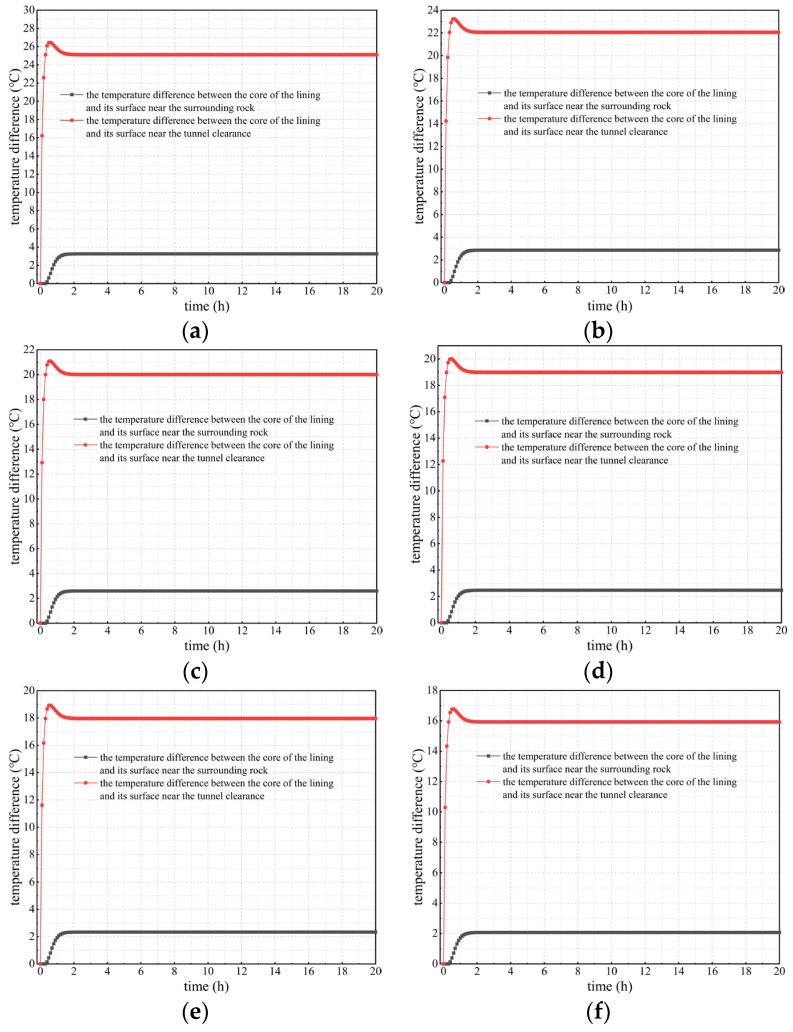
Curves of the temperature difference between the lining’s core and surfaces when not considering hydration heat. (**a**) *T*_outer_ = 95 °C; (**b**) *T*_outer_ = 80 °C; (**c**) *T*_outer_ = 70 °C; (**d**) *T*_outer_ = 65 °C; (**e**) *T*_outer_ = 60 °C; (**f**) *T*_outer_ = 50 °C.

Taking no account of the heat release of concrete, the temperature variation in lining concrete at different positions and times was analyzed, in addition to the temperature difference between the lining core and its surfaces. Calculation results under different calculation conditions when not considering concrete hydration heat are shown in Figure 8, Figure 9 and Figure 10.

1. Spatiotemporal variation in the lining’s temperature field

Figure 8 presents the spatiotemporal variation in the lining’s temperature field under each calculation condition when not considering concrete hydration heat. On the whole, the spatiotemporal evolution of the temperature field of secondary lining under various calculation conditions is similar to that when considering concrete hydration heat, that is the temperature of secondary lining closer to the tunnel clearance decreases faster. Additionally, the range of the strong changing zone of the lining temperature is basically the same, namely the temperature of secondary lining within a thickness range of 15 cm adjacent to the tunnel clearance decreases most drastically. However, when considering concrete hydration heat, overall, the lining temperature is approximately 1–2 °C higher than that when not considering concrete hydration heat after the temperature field reaches stability.

2. Temperature variation in secondary lining under different temperatures and locations

Figure 9 depicts the changing curves of the lining temperature at various temperatures and positions when not considering concrete hydration heat. Seen from the comparative analysis of Figure 6 and Figure 9, the temperature changes in secondary lining at different positions and different external surface temperatures under various calculation conditions are similar. That is, the temperature of secondary lining within a thickness range of approximately 15 cm adjacent to the tunnel clearance decreases most severely. Moreover, the higher the outer surface temperature of secondary lining, the more severely the temperature decreases. However, when concrete hydration heat is not taken into account, the lining temperature basically reaches stability within 2 h after falling, and the temperature change curve after stabilization is a horizontal line. Obviously, this is different to when the lining temperature increases to a certain extent after a sudden drop and reaches stability after approximately 20 h when considering concrete hydration heat.

3. Variation in the temperature difference between the lining’s core and surfaces

Figure 10 shows the curves of the temperature difference between the lining’s core and inner and outer surfaces under various calculation conditions when not considering concrete hydration heat. By comparing and analyzing Figure 7 and Figure 10, considering concrete hydration heat or not, the curves of the temperature difference mentioned above are similar under each calculation condition. The temperature difference between the lining’s core and outer surface is negatively correlated with the outer surface temperature of secondary lining, and it is maintained at 2–3 °C under each calculation condition. In addition, the temperature difference between the lining’s core and inner surface is positively correlated with the outer surface temperature of secondary lining. However, when not considering concrete hydration heat, the temperature difference between the lining’s core and surfaces basically stabilizes within 2 h after rising, and the curve showing the variation in the temperature difference after stabilization is a horizontal line. Obviously, this is different to when the temperature difference between the lining’s core and inner surface gradually decreases after a sharp increase of 2 h and reaches stability after approximately 20 h when considering hydration heat.

When comparing Figure 7 and Figure 10, the temperature difference between the lining’s core and outer surface is maintained at 2–3 °C under each calculation condition, and does not exceed 20 °C. However, there exists the unfavorable situation where the temperature difference between the lining’s core and inner surface exceeds 20 °C. So, during the curing period of secondary lining, there is no need to focus on controlling the temperature difference between the lining’s core and outer surface. Targeted technologies and measures should be adopted to effectively control the temperature difference between the lining’s core and inner surface. In addition, when considering hydration heat, the temperature difference between the lining’s core and inner surface decreases by approximately 1 °C compared to when not considering hydration heat, indicating that hydration heat can reduce the lining’s temperature difference to a certain extent, which has certain benefits for the curing of lining concrete.

In summary, when considering concrete hydration heat, the overall temperature of the tunnel lining is 1–2 °C higher than that when the hydration heat is not considered, and the peak temperature and the temperature at equilibrium also relatively increase. In addition, when considering concrete hydration heat, the possibility of the temperature difference between the lining core and its surfaces exceeding the maximum 20 °C specified in the standards increases, leading to an increased risk of cracking in the tunnel lining, which has an adverse impact on the durability of the lining structure. For high water temperatures, as the water temperature increases, the overall temperature of the tunnel lining gradually increases (including the peak temperature and the temperature after reaching stability). Additionally, the higher the water temperature, the more drastically the temperature of secondary lining within a thickness of approximately 15 cm near the tunnel clearance decreases, which gradually increases the probability of the temperature difference between the core of the tunnel lining and its surfaces exceeding 20 °C.

## 4. Conclusions

By comprehensively adopting a theoretical analysis method and a comparative analysis of numerical examples, the spatiotemporal evolution law of the lining’s temperature field in tunnels was studied considering concrete hydration heat. The following are the major conclusions in this study:

(1) Discrete equations for the temperature field in the unsteady state of secondary lining considering concrete hydration heat were finally established by deducing the discrete equations for the internal nodes and the boundary nodes of secondary linings. Moreover, the solution method for discrete equations was given. The results can lay a theoretical foundation for revealing the spatiotemporal evolution law of the lining’s temperature field considering concrete hydration heat. Additionally, the results also provide certain theoretical support for the reasonable curing of the lining concrete.
(2) Results show that the temperature of secondary lining within a thickness range of approximately 15 cm adjacent to the tunnel clearance decreases most sharply. The higher the outer surface temperature of secondary lining, the more drastically the temperature decreases. The large temperature change can cause a drastic change in the temperature difference in tunnel linings, which is extremely detrimental to the lining concrete’s growth. Additionally, when considering concrete hydration heat, overall, the lining temperature after the temperature field reaches stability is approximately 1–2 °C higher than that when not considering concrete hydration heat.
(3) When considering concrete hydration heat, the lining temperature basically stabilizes within 2 h after dropping, and the temperature change curve after stabilization is a horizontal line. However, when considering concrete hydration heat, the temperature of secondary lining rises to a certain extent after a sudden drop and reaches stability after approximately 20 h. The reason for this is that the heat of the lining near the tunnel clearance is rapidly lost under the action of the cooling measures such as ventilation, and the contribution of hydration heat to the changes in the lining temperature gradually becomes prominent.
(4) The temperature difference between the lining’s core and outer and inner surfaces is negatively and positively correlated with its outer surface temperature, respectively. When the outer surface temperature of tunnel linings is 95, 80, or 70 °C, the temperature difference between the lining’s core and inner surface wholly or partially exceeds 20 °C. However, when the outer surface temperature does not exceed 65 °C, the temperature difference is less than 20 °C. Thus, insulation measures should be taken to control it so as not to exceed 65 °C, preventing problems such as lining cracks caused by an excessive temperature difference.

Although this study analyzed the spatiotemporal changes in the temperature field of high-water-temperature tunnel linings while considering concrete hydration heat, the calculation model was simplified to a circular shape. In future research, modeling and calculation will be carried out based on the actual shape of the tunnel. This study is applicable to the analysis of the temperature field in the lining of high-ground-temperature tunnels, laying the foundation for the analysis of lining temperature stress and providing guidance for the maintenance of high-ground-temperature tunnel linings.

## Figures and Tables

**Table 1:** Summary of symbols in the equations.

Symbols	The Meaning of Symbols in the Equations
*T*	The object temperature (°C), which is a function with variables *x*, *y*, *z* and *τ*.
*τ*	Time of heat conduction (s).
*α*	Thermal diffusivity (m^2^/s), $\alpha =\frac{\lambda }{\rho c}$.
*λ*	Thermal conductivity (W/m·°C).
*ρ*	Medium density (kg/m^3^).
*c*	Specific heat of the medium (J/kg·°C).
$\overset{•}{\Phi }$	Internal heat source (source term), which refers to the energy released in unit time of the microelement.
*T_k_*	Temperature of the *k*-th layer of the lining (°C).
*α_k_*	Thermal diffusivity of the *k*-th layer of the lining (m^2^/s), $\alpha_{k}=\frac{\lambda_{k}}{\rho_{k}c_{k}}$.
*λ_k_*	Thermal conductivity of the *k*-th layer of the lining (W/m·°C).
*ρ_k_*	Density of the *k*-th layer of the lining (kg/m^3^).
*c_k_*	Specific heat of the *k*-th layer of the lining (J/kg·°C).
${\overset{•}{\Phi }}_{k}$	Internal heat source of the *k*-th layer of the lining.
*r*	Radius of the *k*-th layer of the medium (m).
***n***	Vector on the outer surface.
*T_f_*	Fluid temperature (air temperature in the tunnel clearance) (°C).
*h*	Convective conversion coefficient (w/(m^2^·k)).
Δ*r*	Spatial steps (m).
Δ*τ*	Temporal steps (°C).
*F_O_*	Fourier number of grid. It is a dimensionless quantity used to describe the unsteady heat conduction and molecular diffusion, $F_{O}=\frac{\alpha \Delta \tau }{\Delta r^{2}}$.
*r_n_*	Vertical length from node (*n*, *i*) to the longitudinal axis of the tunnel (unit: m), *r_n_* = (*n* − 1)Δ*r*.
$T_{n}^{i}$	Temperature at node (*n*, *i*), K.
*B_i_*	Resistance to the heat conduction ratio (Biot number), $B_{i}=\frac{2h\Delta \tau }{\rho c\Delta r}$.
*r*_1_	Vertical length from the inner boundary to the tunnel’s longitudinal axis (unit: m).
*T*_outer_	The temperature field of the lining’s outer boundary (°C).

**Table 2:** The working conditions.

Position	Thickness (cm)	Computing Time (h)	Whether Concrete Hydration Heat Is Considered	Temperature on the Lining’s Outer Boundary (°C)					
Secondary lining	35 cm	20	No	95	80	70	65	60	50
	35 cm	20	Yes	95	80	70	65	60	50

**Table 3:** Thermophysical parameters of different media.

Parameters	Density (*ρ*, kg/m^3^)	Specific Heat (*c*, j/(kg·k))	Heat Conductivity (*λ*, w/(m·k))	Convective Conversion Coefficient (*h*, w/(m^2^·k))	Thermal Diffusivity (*α*, 10^−6^ m^2^/s)
Secondary lining	2000	960	2.944	35	1.636
Air	1.134	/	0.023	/	/

## Data Availability

Data are contained within the article.
